# Case Report: Pathological complete response yet early brain relapse in HER2-positive breast cancer: a case-based review

**DOI:** 10.3389/fimmu.2025.1668995

**Published:** 2026-01-05

**Authors:** Jing Feng, Yujun Tong, Zhen Zhang, Yuanli He

**Affiliations:** 1 Department of Breast Center, Mianyang Central Hospital, School of Medicine, University of Electronic Science and Technology of China, Mianyang, Sichuan, China; 2 Department of Dermatology, Mianyang Central Hospital, School of Medicine, University of Electronic Science and Technology of China, Mianyang, Sichuan, China

**Keywords:** blood-brain barrier, brain metastases, case report, HER2-positive breast cancer, pathological complete response

## Abstract

Despite advances in anti-HER2 therapies leading to high pathological complete response (pCR) rates, the blood-brain barrier (BBB) still shelters micrometastatic deposits, so intracranial relapse continues to pose a formidable therapeutic obstacle in HER2-positive breast cancer (BC). Understanding the mechanisms underlying early central nervous system (CNS) relapse and integrating BBB-penetrant strategies remain urgent unmet needs. We report a 60-year-old woman with HER2-positive, hormone receptor-negative breast cancer who achieved pCR after neoadjuvant docetaxel combined with trastuzumab and pertuzumab, followed by 12 months of maintenance trastuzumab and pertuzumab. Despite achieving pCR and comprehensive systemic control, the patient developed multifocal brain metastases two months after completing maintenance therapy, without extracranial recurrence. This case underscores the limitations of large-molecule monoclonal antibodies in preventing CNS recurrence due to poor BBB permeability, allowing dormant CNS-adapted clones to persist and later expand. Emerging CNS-active therapies, including small-molecule tyrosine kinase inhibitors (TKIs) such as tucatinib and next-generation antibody-drug conjugates (ADCs) like trastuzumab deruxtecan, have shown promising intracranial activity. In addition, advanced strategies such as intensified MRI surveillance, radiomics, liquid biopsy, focused ultrasound-mediated BBB disruption, nanoparticle delivery systems, and radionuclide therapy offer potential avenues for early identification and prevention of cerebral metastases.

## 1 Introduction

Breast cancer (BC) overexpressing human epidermal growth factor receptor 2 (HER2) constitute roughly 15–20 % of all cases and display a markedly aggressive phenotype, historically associated with poor outcomes (1). Although the advent of HER2 directed therapies has markedly enhanced overall survival rates, between 30 % and 50 % of individuals with HER2-positive BC ultimately experience intracranial metastatic spread, rendering central nervous system (CNS) progression a leading contributor to illness and death in this patient population (2–4).

Neoadjuvant therapy with dual HER2 blockade using trastuzumab and pertuzumab in combination with chemotherapy has significantly increased pathological complete response (pCR) rates to approximately 45–60%, leading to substantial gains in 5-year disease-free survival (DFS). Consequently, this regimen has been widely adopted in international guidelines as the standard of care for high-risk, early-stage HER2-positive BC (5–7). However, large randomized trials, including the APHINITY study, have demonstrated that even with prolonged maintenance therapy using trastuzumab and pertuzumab (HP), the incidence of brain metastases remains largely unchanged, indicating that achieving pCR alone does not fully eliminate the risk of CNS recurrence (8, 9).

Monoclonal antibodies such as trastuzumab and pertuzumab are hydrophilic and are incapable of efficiently crossing an intact blood–brain barrier (BBB). Consequently, these agents can only passively diffuse into CNS lesions after the integrity of the barrier has been compromised by metastatic growth, leading to subtherapeutic drug concentrations within the CNS and the persistent survival of resistant tumor clones (10). To address this therapeutic challenge, novel classes of agents, including small-molecule tyrosine kinase inhibitors (TKIs) and antibody-drug conjugates (ADCs), have been developed to overcome the limitations imposed by the BBB. In the pivotal HER2CLIMB trial, the highly selective HER2-targeted TKI tucatinib reduced the risk of central nervous system (CNS) progression or death by 68% and extended the median CNS progression-free survival (PFS) to roughly 10 months (11). Similarly, trastuzumab deruxtecan (T-DXd), an advanced ADC, achieved a 57–61% intracranial objective response rate (ORR) and maintained overall survival exceeding 90% at 12 months in the DESTINY-Breast 12 study (12).

Despite the demonstrated intracranial activity of new-generation BBB-penetrant agents, optimizing CNS surveillance, identifying high-risk individuals, and prospectively integrating BBB-penetrant therapies in patients with early-stage HER2-positive BC remain unmet clinical needs. Here, we present a case of a patient with HER2-positive BC who developed CNS metastases shortly after completing one year of HP maintenance therapy, despite having achieved a pCR following neoadjuvant treatment. Through a comprehensive review of the current literature, we discuss the advantages and limitations of large-molecule antibodies compared to small-molecule TKIs and ADCs in the prevention and management of CNS recurrence, with the aim of providing insights to guide future early intervention and precision prevention strategies targeting brain metastases in this high-risk patient population.

## 2 Case presentation

### 2.1 Patient information

A 60-year-old postmenopausal woman with no significant past medical history and no known family history of breast or ovarian cancer presented in July 2023 with a palpable mass in the upper-outer quadrant of the right breast, accompanied by nipple inversion and yellow, turbid nipple discharge. Her ECOG performance status was 0 at presentation.

### 2.2 Clinical findings

On physical examination, the right breast exhibited peau d’orange changes and nipple retraction. Multiple firm, mobile lymph nodes were palpable in the ipsilateral axillary and right supraclavicular regions.

### 2.3 Diagnostic assessment

**Figure 1:**
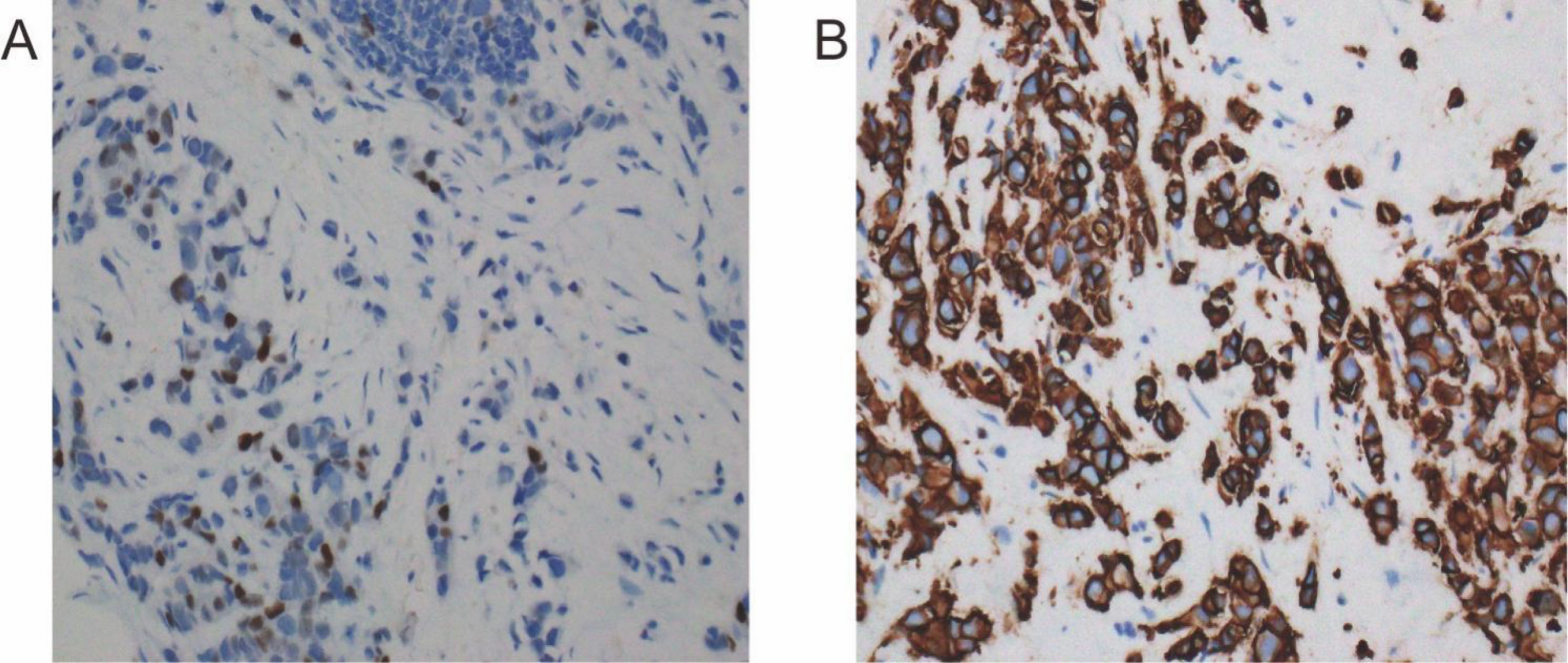
Baseline immunohistochemistry of the diagnostic core-needle biopsy (×200). **(A)** Estrogen-receptor staining shows <1% positive nuclei (ER-negative). **(B)** HER2 staining demonstrates uniform, intense (3+) membranous reactivity in >10% of tumor cells, confirming HER2 over-expression.

Breast MRI identified an irregular 4.3 × 3.6 cm lesion with several satellite nodules in the superolateral segment of the right breast, accompanied by enlargement of ipsilateral axillary and supraclavicular lymph nodes. Ultrasound-guided core biopsy verified invasive breast carcinoma of no special type (NST) and assigned WHO grade 2 (aggregate score 6/9: tubule formation 3, nuclear atypia 2, mitotic activity 1). Immunohistochemical analysis showed absent estrogen receptor (ER) and progesterone receptor (PR) expression, weak-to-moderate androgen receptor (AR) positivity in 40 % of tumor cells, strong HER2 staining (3+), and a Ki-67 proliferation index of 25 %. **Figure 1** presents representative ER and HER2 immunoreactivity.

Ultrasound-guided fine-needle aspiration of an ipsilateral axillary node identified a secondary NST carcinoma with similar receptor status (ER 3% weak positive, PR negative, HER2 3+, Ki-67 25%). Cytology of a right supraclavicular node revealed clusters of pleomorphic malignant cells, consistent with metastatic carcinoma. At the time of diagnosis, the patient reported no neurologic symptoms, including headache, visual disturbance, focal weakness, sensory change, or gait instability. Baseline systemic staging with contrast-enhanced computed tomography of the chest, abdomen, and pelvis, as well as a bone scan, showed no evidence of visceral or skeletal metastases. However, no baseline brain CT or MRI was obtained, so the presence of occult CNS disease at presentation cannot be definitively excluded. According to the AJCC 8th edition, the clinical stage was cT4b cN3c M0 (Stage IIIC).

### 2.4 Therapeutic interventions

**Figure 2:**
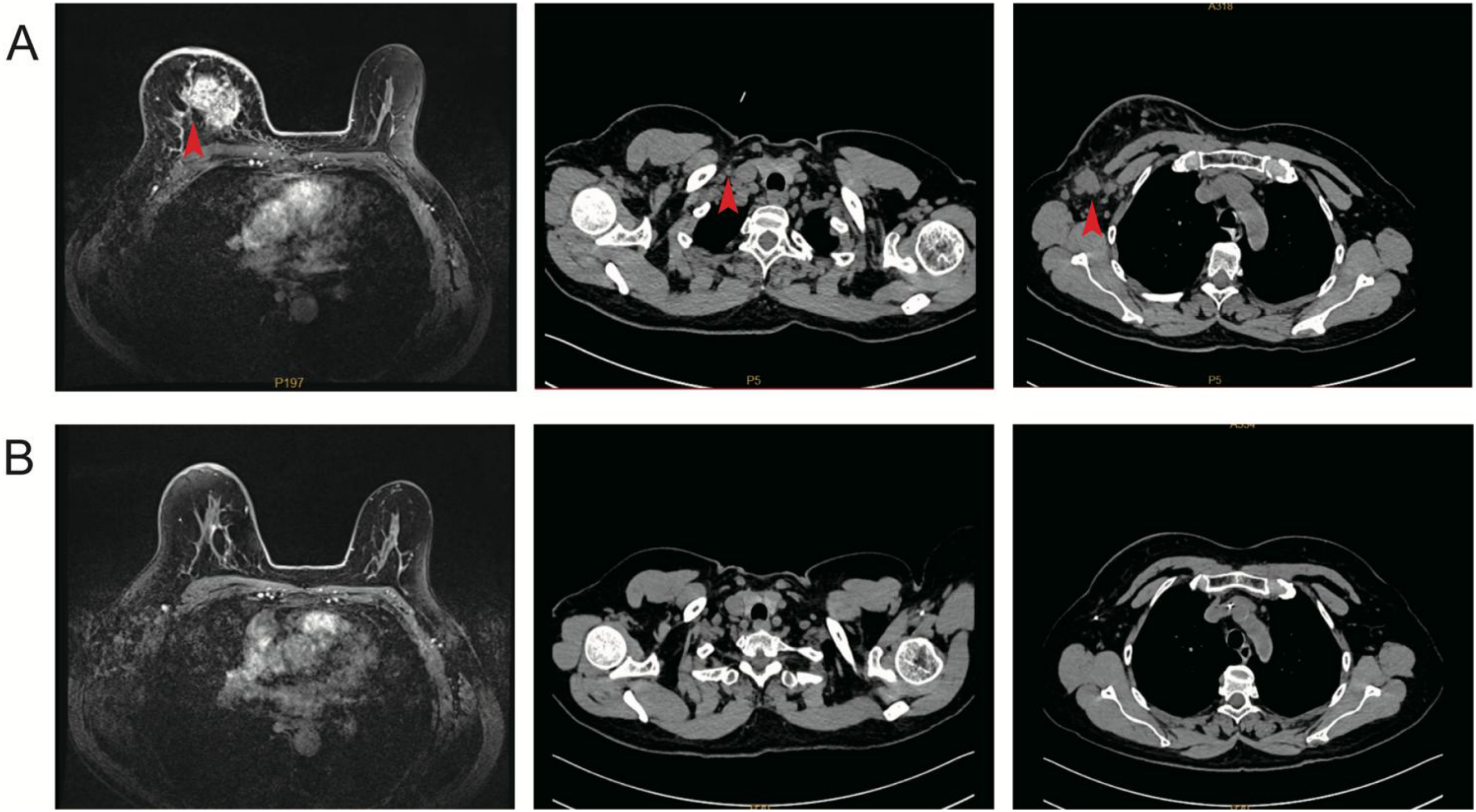
Imaging response to neoadjuvant THP therapy. **(A)** Pre-treatment breast MRI displays a 4.3 × 3.6 cm irregular mass with satellite foci; chest CT shows enlarged right axillary and supraclavicular lymph nodes. **(B)** Post-treatment MRI (after four cycles) reveals near-complete resolution of the primary lesion, and follow-up CT documents regression of nodal disease.

Neoadjuvant therapy (02 August 2023–08 October 2023): The patient received four cycles of docetaxel, trastuzumab and pertuzumab on every 3 week cycle. Treatment was well tolerated overall; the main toxicities were grade 1–2 alopecia and fatigue without febrile neutropenia or treatment delays. Interim MRI after cycle 4 demonstrated marked tumor regression and resolution of nodal disease (radiologic partial response). Pre- and post-neoadjuvant imaging are compared in **Figure 2**.

Surgery (01 November 2023): Given the initial bulky, biopsy-proven supraclavicular and lower cervical nodal involvement (cN3c) in the absence of distant metastases, the case was reviewed at a multidisciplinary tumor board comprising breast surgeons, head-and-neck surgeons, medical oncologists, and radiation oncologists. The team recommended a combined right modified radical mastectomy with level I–III axillary dissection and right cervical nodal clearance as an individualized approach to optimize locoregional control and obtain precise pathological staging. We acknowledge that cervical lymph node dissection is not a standard component of curative-intent surgery for breast cancer and should be considered only on a case-by-case basis. The patient underwent an modified radical mastectomy on the right side with level I–III axillary dissection and right cervical nodal clearance. The postoperative course was uneventful, and the patient was discharged on postoperative day 7 without surgical complications such as wound infection or lymphedema.

**Figure 3:**
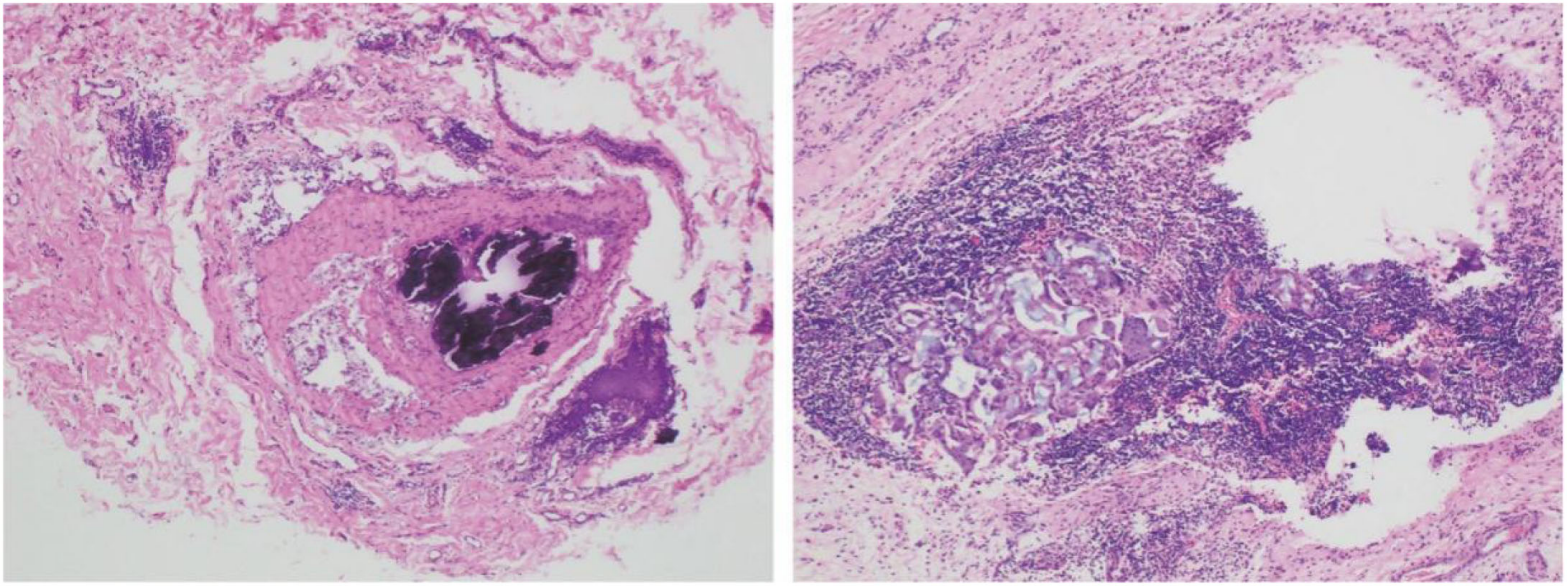
Post-mastectomy H&E section (×100) demonstrating a fibrotic tumor bed with granulomatous–lymphoid reparative reaction and no residual invasive carcinoma (Miller–Payne grade 5, pCR).

Pathology: No residual invasive carcinoma was identified in the breast (Miller–Payne grade 5; pCR). All axillary lymph nodes (n = 10) and cervical level III–V lymph nodes (n = 23) were negative for metastasis, and all surgical margins were clear. Representative histology is shown in **Figure 3**. The final pathological stage was ypT0N0.

Adjuvant systemic therapy: 21 December 2023–21 February 2024: Four 3-weekly courses of doxorubicin (60 mg/m²) in combination with cyclophosphamide (600 mg/m²). No dose reductions were required during AC, and no clinically significant cardiac events were observed. 14 March 2024–19 November 2024: Maintenance therapy with trastuzumab plus pertuzumab every 3 weeks for 13 cycles (total anti-HER2 therapy duration: 1 year). Serial echocardiography demonstrated a consistent left ventricular ejection fraction ≥ 60%.

**Figure 4:**
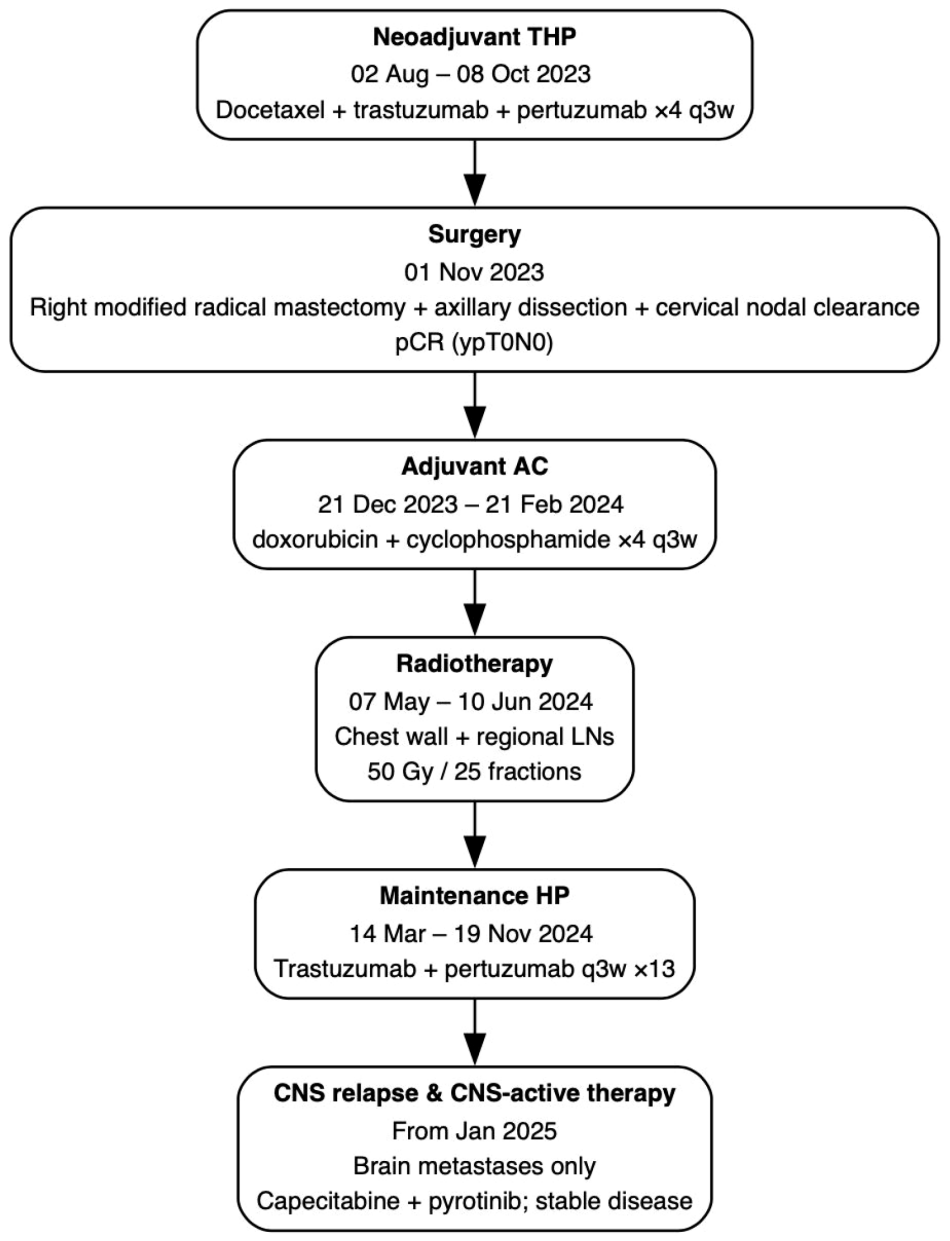
Treatment timeline summarizing systemic and locoregional therapies from initial diagnosis to CNS relapse.

Radiotherapy (07 May 2024–10 June 2024): External beam irradiation to the chest wall and regional lymph node basins (50 Gy in 25 fractions). A detailed chronology of all systemic and locoregional treatments—from neoadjuvant therapy through adjuvant phases to management of CNS relapse—is provided in **Figure 4**.

### 2.5 Follow-up and outcomes

The patient remained disease-free until January 2025 when she developed persistent occipital headaches. A brain CT scan performed at an outside facility revealed an enhancing lesion consistent with brain metastases, while restaging CT showed no extracranial recurrence. The ECOG performance status was 1.

**Figure 5:**
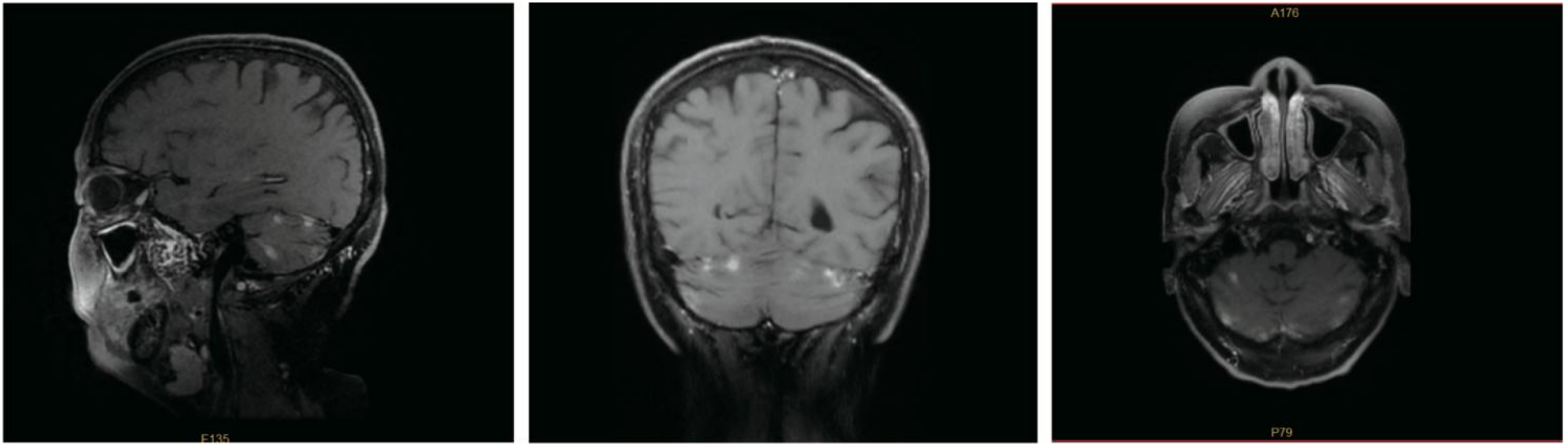
Contrast-enhanced brain MRI on 06 Apr 2025 showing multifocal enhancing lesions in both cerebellar hemispheres, consistent with brain metastases.

On 15 January 2025, systemic therapy was switched to oral pyrotinib given once each day at a 400 mg dose, combined with capecitabine dosed at 1.0 g/m² twice daily on days 1–14 of every 21-day cycle. The only adverse event noted was grade 1 hand-foot syndrome. After six weeks of therapy, a brain MRI performed at our institution on 06 April 2025 demonstrated stable disease without new lesions (**Figure 5**). A multidisciplinary team recommended stereotactic radiosurgery to the residual cerebellar foci and consideration of trastuzumab deruxtecan upon progression; however, the patient currently declined radiotherapy.

At the last presubmission follow-up in May 2025, the patient was alive with controlled neurologic symptoms and preserved quality of life (ECOG 1). At the most recent follow-up in November 2025, she remained alive without new neurologic symptoms and continued on pyrotinib plus capecitabine with stable intracranial disease.

## 3 Discussion

This case illustrates a clinically important pattern of failure in contemporary HER2-positive breast cancer: despite pCR after neoadjuvant dual HER2 blockade and completion of 1 year of trastuzumab plus pertuzumab, the CNS became the first and only site of relapse. The most plausible explanation is that the BBB and the CNS tumor microenvironment together create an anatomic and immunologic sanctuary in which HER2-positive subclones can survive and later expand under minimal therapeutic pressure.

From a pharmacologic perspective, the BBB is highly restrictive to large hydrophilic molecules. Trastuzumab and pertuzumab, with molecular weights of approximately 148 kDa, are essentially unable to cross an intact BBB (13). Preclinical investigations indicate that, when the blood–brain barrier is intact, trastuzumab’s cerebrospinal fluid exposure amounts to only 0.01–0.03 % of the plasma AUC, and zero-flow microdialysis further shows brain interstitial concentrations reaching merely 0.3–0.5 % of systemic levels, indicating severely inadequate therapeutic concentrations within the CNS (14, 15). Consequently, even when complete remission is achieved in extracranial lesions with dual HER2 blockade, micrometastatic foci within the brain may persist in a low-drug-pressure environment, allowing for prolonged dormancy and gradual outgrowth (16). Under conditions of strong systemic disease control, resistant clones or CNS-adapted subclones may gain a relative growth advantage, making intracranial lesions the earliest or sole manifestation of relapse (17). Multicentre retrospective series indicate that, among women with HER2-enriched breast carcinoma who receive preoperative trastuzumab plus pertuzumab, roughly 1–3% percent develop intracranial metastasis as the earliest and sole site of relapse within two years, independent of pCR status (18). Additionally, brain metastases exhibit specific resistance characteristics, including astrocyte-mediated IL-6/STAT3 inflammatory signaling, lipid metabolic reprogramming, and an immunosuppressive microenvironment, resulting in significantly lower sensitivity to existing antibody therapies compared to extracranial lesions (19–21).

These observations have catalyzed the development of BBB-penetrant HER2-directed therapies. Among TKIs, tucatinib is notable for achieving a free brain-to-plasma concentration ratio (Kp,uu,brain) of up to 0.65 under intact BBB conditions, markedly higher than lapatinib or neratinib (22). In the phase three HER2CLIMB trial, adding tucatinib to trastuzumab plus capecitabine lowered the hazard of central nervous system progression or mortality by 68% in patients with active brain lesions, increasing median intracranial PFS from 4.2 to 9.9 months (11). Building on these data, the ongoing single-arm, phase II BRIDGET trial (NCT05323955) is evaluating the addition of tucatinib to maintenance trastuzumab/pertuzumab or T-DM1 after local therapy for isolated brain metastases in patients with advanced HER2-positive breast cancer (23) By enrolling patients with stable extracranial disease after stereotactic radiosurgery and/or surgical resection, BRIDGET exemplifies a ‘secondary prevention’ strategy that aims to delay further intracranial progression through sustained BBB-penetrant HER2 blockade.

Other TKIs have also demonstrated meaningful CNS activity. In the PERMEATE phase II study, pyrotinib plus capecitabine achieved a 74.6% intracranial objective response and yielded a median intracranial PFS of roughly 11 months in individuals with HER2 positive BC and existing brain metastases who had not yet received cranial radiotherapy, underscoring the capacity of small-molecule HER2 inhibitors to improve CNS disease control (24). In our patient, the combination of pyrotinib and capecitabine achieved at least radiologic stabilization of cerebellar lesions with preservation of performance status, supporting its use as a pragmatic CNS-active regimen in settings where tucatinib is not yet readily available.

ADCs represent a complementary BBB-penetrant strategy, particularly in the context of a disrupted blood–tumor barrier. T-DXd has shown robust intracranial efficacy in multiple studies. In the TUXEDO-1 trial, T-DXd produced a 73.3% intracranial response rate and a median CNS PFS of 14 months in patients with active brain metastases (25). In the phase III DESTINY-Breast03 trial, T-DXd reduced the risk of intracranial progression versus rastuzumab emtansine (T-DM1) by 72% and achieved intracranial responses in 63.9% of patients, compared with 33.3% with T-DM1 (26). More recently, DESTINY-Breast09 demonstrated that first-line T-DXd plus pertuzumab significantly improved PFS compared with taxane, trastuzumab, and pertuzumab in patients with HER2-positive metastatic breast cancer, suggesting that potent HER2-directed ADCs may soon move into the first-line setting and may be particularly attractive for patients at high risk of CNS relapse (27). T-DM1 itself retains clinically relevant CNS activity: in the KAMILLA phase IIIb cohort, an intracranial objective response rate of 42.9% was observed among patients with brain metastases, supporting its use as a post-radiotherapy maintenance option in selected cases (28).

Efforts are now underway to test whether these CNS-active agents can prevent or delay brain metastases in earlier-stage disease. The phase III CompassHER2-RD (A011801) trial is evaluating tucatinib plus T-DM1 in patients with residual HER2-positive disease after neoadjuvant therapy, with particular interest in late CNS events (29). DESTINY-Breast05 is comparing adjuvant T-DXd with T-DM1 in a similar high-risk population and uniquely includes “time to brain metastasis” as a key exploratory endpoint (30). Additionally, the TRAIN-4 phase Ib study is exploring a chemotherapy-free neoadjuvant regimen of preoperative tucatinib combined with trastuzumab and pertuzumab, aiming to reduce subsequent brain metastatic spread while potentially minimizing systemic toxicity (31). Collectively, these trials move the field toward a paradigm in which BBB-penetrant TKIs and ADCs may be incorporated earlier in the disease course for patients with biologic features that confer a high likelihood of CNS relapse.

Our case also aligns with known biologic risk factors for brain metastasis. Retrospective studies indicate that ER/PR-negative, HER2-positive subtypes carry a markedly higher 5-year cumulative incidence of CNS involvement than other molecular phenotypes (32). Additional risk markers include high proliferative index (Ki-67 ≥ 30%) and axillary nodal positivity, both of which can independently double the 3-year risk of brain metastasis (33, 34). Mechanistically, loss of estrogen and progesterone signaling promotes epithelial–mesenchymal transition and anoikis resistance, enhancing migratory capacity and survival of detached tumor cells (35, 36). Persistent HER2 hyperactivation sustains PI3K–AKT and RAS–MAPK signaling, thereby facilitating invasion, intravascular survival, and early generation of circulating tumor cells. Experimental models implicate the adaptor protein GRB2, together with the β4-integrin/SRC–FAK complex, as a critical mechanical mediator that promotes transendothelial migration of HER2-positive cells across the BBB (37). Brain-tropic clones further upregulate molecules such as L1CAM, COX-2, HB-EGF, cathepsin S and ST6GALNAC5, which weaken endothelial junctions and enhance adhesion and transmigration into the CNS parenchyma (38). Once established in the brain, tumor cells release exosomes enriched in miR-105, miR-181c and Annexin A2 that reprogramme astrocytes (39, 40). These reprogrammed astrocytes secrete IL-6 and IL-1β, activating JAK2/STAT3 signaling in tumor cells and enabling dynamic switching between dormancy and proliferation within the CNS niche (41). Approximately 40% of breast cancer brain metastases also express the a AR (42). AR signaling via the AR–PGC1α–OXPHOS axis facilitates metabolic adaptation to the glucose-depleted, oxidative stress-rich brain microenvironment and further supports survival and treatment resistance (43, 44). Together, these pathways underscore that HER2-positive brain metastasis is the end result of both systemic dissemination and highly specific immunologic and metabolic adaptation to the CNS microenvironment.

Advances in risk stratification and surveillance may help identify patients who would benefit from earlier CNS-directed strategies. Cross-tissue transcriptomic association analyses have identified a 12-gene signature, including CASP8, that distinguishes primary tumors with high brain-metastatic potential. models based on this signature achieved a concordance index exceeding 0.80 in external validation cohorts (45). Weighted gene co-expression network analyses highlight key immune-regulatory genes such as THY1 as being strongly associated with brain metastases, suggesting that early immune evasion may accelerate CNS dissemination (46). Radiomics models developed using preoperative conventional MRI have also demonstrated robust predictive capability, with an area under the curve of 0.88 for forecasting the risk of brain metastasis within two years, significantly outperforming traditional clinical subtyping (47). A prospective phase II trial demonstrated that screening brain MRI in metastatic BC patients without neurologic symptoms identified silent brain metastases in 14% of cases at baseline. Follow-up imaging at six months increased the cumulative detection rate to 24%, highlighting the importance of enhanced radiologic monitoring for timely identification of intracranial involvement (48). A recent international expert consensus panel reported that approximately 70% of experts supported incorporating baseline and serial brain MRI into surveillance for neurologically asymptomatic patients with HER2-positive or triple-negative metastatic breast cancer; however, this proposal did not reach formal consensus and routine screening MRI remains controversial (49). Furthermore, liquid biopsy technologies have further enhanced the sensitivity of detecting brain metastases; circulating tumor DNA in cerebrospinal fluid has been shown to identify 83% of driver mutations associated with brain metastases, compared to only 28% detection in matched plasma samples, enabling molecular relapse signals to be identified several months prior to radiographic progression (50). In parallel, circulating exosomal microRNAs, such as miR-10b and miR-20b, are emerging as promising biomarkers and potential therapeutic targets for predicting and managing BC brain metastases (51). Our patient did not undergo baseline brain imaging or CNS-specific liquid biopsy at diagnosis, highlighting both current practice gaps and opportunities for more proactive risk-adapted CNS assessment in future.

Given the clinical challenge posed by the poor permeability of large-molecule agents across an intact BBB in HER2-positive BC brain metastases, emerging BBB-penetrating drug delivery technologies are actively being explored in clinical settings. Clinical studies have demonstrated that focused ultrasound combined with microbubbles can temporarily and reversibly disrupt the local BBB, significantly enhancing the intraparenchymal concentration of large-molecule agents such as trastuzumab, with a favorable safety profile and repeatability, thereby showing promising potential for clinical translation (52, 53). Additionally, nanoparticle-based delivery systems that exploit receptor-mediated transcytosis, active tumor targeting and enhanced permeability and retention can markedly increase drug uptake and antitumor activity in brain metastasis models (54, 55). Moreover, targeted radionuclide therapies, such as ^225Ac-DOTA-trastuzumab, despite limited absolute uptake in brain tissue, can achieve significant tumoricidal effects in brain metastasis models due to their high linear energy transfer properties, providing a novel “high-energy, low-dose” therapeutic approach (56). In the realm of theranostics, HER2-targeted small-molecule probes, such as ^68Ga-labeled HER2 nanobodies and affibodies, have entered early-phase clinical trials, potentially enabling precise imaging and targeted treatment of brain metastases in the future (57). These strategies based on physics, nanotechnology and nuclear medicine offer complementary avenues for overcoming the BBB and could be integrated with CNS-active TKIs and ADCs in future combinatorial approaches.

This report has several limitations. First, it describes a single patient and therefore cannot be generalized to all individuals with HER2-positive breast cancer; the case should be regarded as illustrative. Second, no baseline brain CT or MRI was obtained at the time of diagnosis. Although the patient had no neurologic symptoms and conventional staging with CT and bone scan excluded extracranial distant metastases, occult CNS involvement cannot be definitively ruled out, and the apparently early CNS relapse may in part reflect progression of subclinical brain metastases. Third, some management decisions in this case—notably the use of cervical lymph node dissection within curative-intent surgery—were made as individualized, multidisciplinary choices and may not be directly applicable in all practice settings.

## 4 Conclusion

This case underscores the persistent risk of CNS metastasis in individuals with HER2-positive BC, even after achieving pCR following neoadjuvant dual HER2 blockade and completing one year of maintenance therapy with HP. The BBB continues to serve as an anatomical sanctuary that limits effective drug delivery, allowing for the survival and proliferation of CNS-adapted tumor clones. Emerging evidence highlights the potential of BBB-penetrant therapies, including small-molecule TKIs and ADCs, in improving intracranial disease control. However, optimizing CNS surveillance strategies, identifying individuals at high risk for CNS relapse, and determining the appropriate timing for the integration of CNS-active agents remain unmet clinical needs in the management of early-stage HER2-positive BC. Further prospective studies are warranted to evaluate preventive strategies targeting brain metastasis, with the goal of improving long-term survival and quality of life in this high-risk patient population.

## Data Availability

The original contributions presented in the study are included in the article/**Supplementary Material**, Further inquiries can be directed to the corresponding author/s.
