# Supplementary material for: Case Report: Pathological complete response yet early brain relapse in HER2-positive breast cancer: a case-based review
Source: Front Immunol. 2026 Jan 5;16:1668995. doi: 10.3389/fimmu.2025.1668995 (PMC12813026; doi:10.3389/fimmu.2025.1668995)
Supplement: Supplementary file 1 [file DataSheet1.pdf]

CARE case report guidelines

CARE Checklist of information to include when writing a case report

BY-NC-ND

Topic	Item	Checklist item description	Reported on Line
Title	1	The diagnosis or intervention of primary focus followed by the words "case report"	0
Key Words	2	2 to 5 key words that identify diagnoses or interventions in this case report, including "case report"	14-15
Abstract (no references)	3a	Introduction: What is unique about this case and what does it add to the scientific literature?	19-21
	3b	Main symptoms and/or important clinical findings	26-27
	3c	The main diagnoses, therapeutic interventions, and outcomes	23-25
	3d	Conclusion—What is the main "take-away" lesson(s) from this case?	27-30
Introduction	4	One or two paragraphs summarizing why this case is unique (**may include references**)	44-73
Patient Information	5a	De-identified patient specific information.	76
	5b	Primary concerns and symptoms of the patient.	77-78
	5c	Medical, family, and psycho-social history including relevant genetic information	76
	5d	Relevant past interventions with outcomes	98-116
Clinical Findings	6	Describe significant physical examination (PE) and important clinical findings.	80-91
Timeline	7	Historical and current information from this episode of care organized as a timeline	511-514
Diagnostic Assessment	8a	Diagnostic testing (such as PE, laboratory testing, imaging, surveys).	84-96
	8b	Diagnostic challenges (such as access to testing, financial, or cultural)	NA
	8c	Diagnosis (including other diagnoses considered)	87
	8d	Prognosis (such as staging in oncology) where applicable	96
Therapeutic Intervention	9a	Types of therapeutic intervention (such as pharmacologic, surgical, preventive, self-care)	98-116
	9b	Administration of therapeutic intervention (such as dosage, strength, duration)	98-116
	9c	Changes in therapeutic intervention (with rationale)	98-116
Follow-up and Outcomes	10a	Clinician and patient-assessed outcomes (if available)	129-130
	10b	Important follow-up diagnostic and other test results	124-126
	10c	Intervention adherence and tolerability (How was this assessed?)	126-128
	10d	Adverse and unanticipated events	124
Discussion	11a	A scientific discussion of the strengths AND limitations associated with this case report	66-73
	11b	Discussion of the relevant medical literature **with references**.	132-255
	11c	The scientific rationale for any conclusions (including assessment of possible causes)	132-143
	11d	The primary "take-away" lessons of this case report (without references) in a one paragraph conclusion	257-267
Patient Perspective	12	The patient should share their perspective in one to two paragraphs on the treatment(s) they received	NA
Informed Consent	13	Did the patient give informed consent? Please provide if requested	Yes ☑ No ☐
